# Salvage of Suboptimal or Occluded Arteriovenous Fistulas Using a 4 French System From the Radial Artery for Initial Balloon Angioplasty Maturations

**DOI:** 10.7759/cureus.13446

**Published:** 2021-02-19

**Authors:** Christian Voto, Thomas Panetta

**Affiliations:** 1 College of Osteopathic Medicine, University of New England, Biddeford, USA; 2 Vascular Surgery, Mercy Medical Center, Rockville Centre, USA

**Keywords:** end stage renal disease (esrd), arterio-venous fistula, chronic kidney disease, peripheral vascular surgery, arterio-venous graft, diabetes, suboptimal veins, hemodialysis access, maintenance hemodialysis, 4 french catheters

## Abstract

Introduction

End-stage renal disease (ESRD) is a condition that has seen a large increase in prevalence in recent decades. Paralleling this increase in prevalence is the increasing number of patients requiring vascular access for hemodialysis. Arteriovenous fistulas (AVFs) and arteriovenous grafts (AVGs) are considered the procedures of choice for hemodialysis access. However, due to the suboptimal venous anatomy (<2 mm diameter, sclerotic vascular walls) and chronic medical conditions (diabetes mellitus) seen in many ESRD patients, successful AVF creation and maturation is not always possible using standard procedures. In this study, we performed primary balloon angioplasty (PBA) at the time of AVF creation with subsequent balloon angioplasty maturation (BAM) procedures in a group of patients with a large proportion of diabetes and suboptimal venous anatomy. The purpose of this study was to compare the assisted patency and survival rates in patients with suboptimal veins used to create AVFs to patients with standard vein AVFs and AVGs.

Methods

Over a nine-year period, PBA during AVF creation was performed 682 times. Of these, 551 AVFs were matured in optimally sized veins using standard BAM procedures, and 131 AVFs were matured in suboptimal veins utilizing a modified approach. In the subset of patients with suboptimal venous anatomy, we performed the initial BAM procedure via the radial artery utilizing a 4 French system. Additionally, routine clinical surveillance was scheduled throughout the study period for all patients. Suboptimal veins included small (<2 mm diameter), sclerotic, accessory, or recanalized veins. During the study period, 69 AVGs were created and matured using standard graft-gram procedures. A Kaplan-Meier analysis of survival and assisted patency rates comparing the three groups were calculated utilizing data from a retrospective database and medical records. A hazard ratio and a log-rank test were calculated to assess statistical significance.

Results

The mean time of follow-up for all three groups (n=703) was 43.2 months. Among patients requiring hemodialysis access in the study, the fistula creation rate was 90.8%. Patients with suitable venous anatomy who underwent AVF creation with PBA and standard BAM procedures experienced higher primary assisted patency rates relative to the suboptimal vein AVF and AVG groups (p<0.0001). No difference was seen between the suboptimal vein AVF and AVG groups. Patient survival and the percentage of diabetics were comparable amongst all three groups.

Conclusion

Using our approach, we were able to achieve a high AVF creation rate amongst a group of patients with a large proportion of suboptimal veins and diabetes. Despite not performing as well as standard vein AVFs in regards to primary assisted patency, the patients with suboptimal vein AVFs experienced similar patency and survival rates as compared to patients receiving AVGs. This new approach enhances the ability to create AVFs in patients who would otherwise not be amenable to fistulas and may contribute to reduced complication risk and improved overall survival.

## Introduction

Chronic kidney disease, specifically end-stage renal disease (ESRD), is a disease that affects over 750,000 people in the United States and has seen a 92% increase in prevalence since 2000 [1]. Paralleling the increase in patients with ESRD is the number of patients requiring vascular access for hemodialysis (HD). Among the prevalent ESRD cases as of December 31, 2017, 62.7% were on hemodialysis, which is 84.1% larger than the prevalent hemodialysis population seen in 2000 [1].

The current Kidney Disease Outcome Quality Initiative (KDOQI) guidelines suggest that prevalent HD patients should use either an arteriovenous fistula (AVF) or arteriovenous graft (AVG) for vascular access over a central venous catheter (CVC) due to lower complication rates (infection, thrombosis, and mortality) [2]. Previous initiatives have suggested that when selecting between an AVF and AVG for vascular access, AVFs should be the first option due to reduced cost, higher long-term patency, and lower complication rates [3-4]. However, this preconceived notion that AVFs demonstrate superior patency and lower complication rates as compared to AVGs is no longer supported in the current KDOQI guidelines [2,5]. Several studies comparing vascular access types have been acknowledged by the committee, which has led them to reframe their recommendations. Therefore, a clinician’s first choice of vascular access should not be generalized to all ESRD patients and instead be decided based on individual patient characteristics (vascular anatomy, comorbidities, health circumstances, and patient preference) [2,5].

Similar patency rates among AVFs and AVGs have been reported in the literature [6-7]. In fact, several randomized control trials amongst patients with suboptimal vascular anatomy (<2 mm diameter, sclerotic, recanalized veins) and comorbidities (diabetes mellitus) suggest there may even be a benefit in using AVGs as compared to AVFs [8]. Studies have cited adverse events occurring in patients with AVFs created using suboptimal anatomy. For example, cephalic veins with a diameter smaller than 2.5 mm have been associated with increased immediate failure and decreased primary patency rates [9]. Furthermore, inferior results in forearm AVFs in diabetics have led to recommendations for upper-arm (brachiocephalic) fistulas as the initial fistula choice [10-13].

The methods that are being evaluated in this study specifically address factors that improve the ability to create AVFs in a group of patients with a large proportion of diabetes mellitus and suboptimal venous anatomy. These methods include: 1) Primary balloon angioplasty (PBA) at the time of fistula creation with subsequent balloon angioplasty maturation (BAM) procedures; 2) utilization of radial artery access using a 4 Fr sheath and a low profile 0.018” system for the initial BAM in patients with suboptimal veins; and 3) routinely scheduled clinical surveillance with interventions as needed [14-16]. The purpose of this study was to compare the assisted patency and survival rates in patients with suboptimal veins used to create AVFs to patients with standard vein AVFs and AVGs.

This article was previously presented as a meeting poster at the 2017 Eastern Vascular Society's Annual Conference on October 10, 2017.

## Materials and methods

Surgical techniques

To help increase the number of successful primary AVFs and reduce maturation times, we utilized techniques such as PBA during fistula creation combined with BAM procedures every two to three weeks until the fistula was fully mature and usable for dialysis. Patients with adequately sized veins (>2 mm diameter) underwent PBA with balloons ranging from 2.5 mm to 4 mm in diameter during the surgical procedure and patients with suboptimal veins (<2 mm, sclerotic, accessory, or occluded veins) underwent PBA with balloons ranging from 1.5 mm to 2.5 mm in diameter. The length of the angioplasty segment ranged from 4 cm to 12 cm.

Sequential BAM procedures were performed 2 to 3 mm larger than the measured vein diameter at each previous session. The length of the AVF used for access (venipuncture) was sequentially dilated to an average diameter of 8 mm for forearm AVFs and to 12 to 16 mm for upper arm AVFs. Additional techniques were implemented to further improve the salvage of these suboptimal vein AVFs, including performing micro-puncture access into the radial artery with a 4 Fr sheath and a 0.018” system for the initial recanalization or angioplasty (modified BAMs). Patients were allowed up to two occlusions until the fistulas were abandoned and an alternative site or graft was utilized. Once the inflow or outflow of the fistula was greater than 6 mm in diameter, access for the BAM was transitioned from a 4 Fr radial approach to a standard approach with sheath insertion in the proximal or distal fistula. Permacaths were removed within two weeks of achieving a successful fistula. AVGs were created and maintained utilizing standard graft-gram procedures.

Routine clinical surveillance every three to six months, as well as interventions, if needed, prior to AVF failure were performed in all our patients. Our clinical evaluations involved a thorough physical examination of the fistulas inflow, usable segment, and outflow observing for pulsatility, weakness, reasons for extravasation, and thrill. We also used fistulograms on our patients, as well as intravascular ultrasound, for any patients with a contrast allergy every two to three months to evaluate for fistula patency. Vascular complications that occurred throughout the study period included thrombosis, fistula occlusion, aneurysm formation, steal, and graft infection. These complications were addressed with interventions such as thrombectomy, recanalization, aneurysm resection, coil embolization, and excision of infected grafts.

Study design

Using a large database and chart analysis of patients with ESRD, we looked at patients who received AVFs versus AVGs for primary hemodialysis access and examined their effect on fistula patency and patient survival. Additionally, patients receiving AVFs for primary access were then further subdivided into subsets based on whether they had suboptimal veins and received their initial BAM utilizing a 4 Fr sheath and a low profile 0.018” system for the first and/or second BAM procedures or they had optimal veins and received standard BAM procedures (which utilized sheath insertion in the proximal or distal fistula) every time.

From June 2008 until June 5, 2017, 751 arteriovenous access procedures were performed (682 AVFs and 69 AVGs) in 703 patients. Among the patients receiving fistulas, 551 AVFs in 526 patients were performed in patients with vein diameters > 2 mm and 131 AVFs in 117 patients were performed in patients with suboptimal veins. A total of 69 AVGs were performed in the remaining 60 patients. Additional data were collected on the location of the fistula (radiocephalic vs brachiocephalic) and the proportion of patients with diabetes.

A Kaplan-Meier analysis of survival and assisted patency rates comparing these three groups were calculated utilizing data from medical records and the retrospective database. A hazard ratio was calculated to assess the magnitude of the probable benefit or harm of the treatment compared to the control group and a log-rank test was used to confirm a statistical significance between groups. Differences were considered significant at P values <0.05. Technical success was defined as the ability to successfully utilize the AVF for dialysis.

This study was deemed exempt by the institutional review boards of both Mercy Medical Center and the University of New England (IRB#: 060617-001). Informed consent was obtained by all patients participating in the study. No personal identifying information was included within this article.

## Results

The mean time of follow-up for all three groups (n=703) was 43.2 months. The fistula creation rate amongst patients in this study was 90.8% (682/751). Among the fistulas created, 19.2% (131/682) were done using suboptimal venous anatomy. Time to fistula maturation varied from two to nine months, with the majority being useable in less than three to four months. Among our patients, 43.8% were diabetics (308/703). The percentage of diabetics was 43.4% in the standard vein AVF group, 45.1% in the suboptimal vein AVF group, and 43.2% in the AVG group. Among our patients, 19.6 % (134/682) received brachiocephalic (upper-arm) fistulas, with the remaining undergoing radiocephalic (forearm) fistulas. Differences in patency rates among brachiocephalic and radiocephalic fistulas were not included in this study.

**Figure 1:**
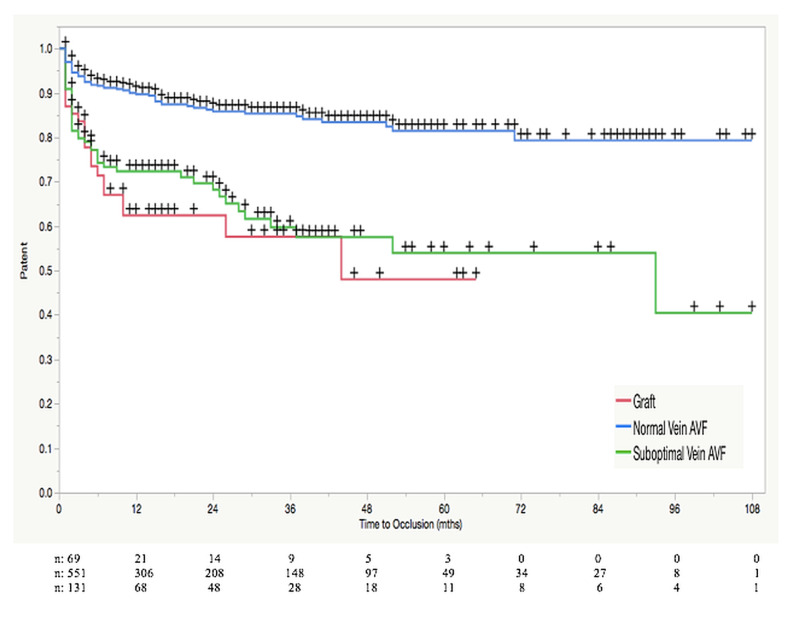
Kaplan-Meier analysis of primary assisted patency rates among patients with various types of hemodialysis access Number of patients at risk (n) with each group is presented in the graph. Patency rates are shown as percentages at various points in time (months). P values calculated with the log-rank test. + signifies censored data point.

**Figure 2:**
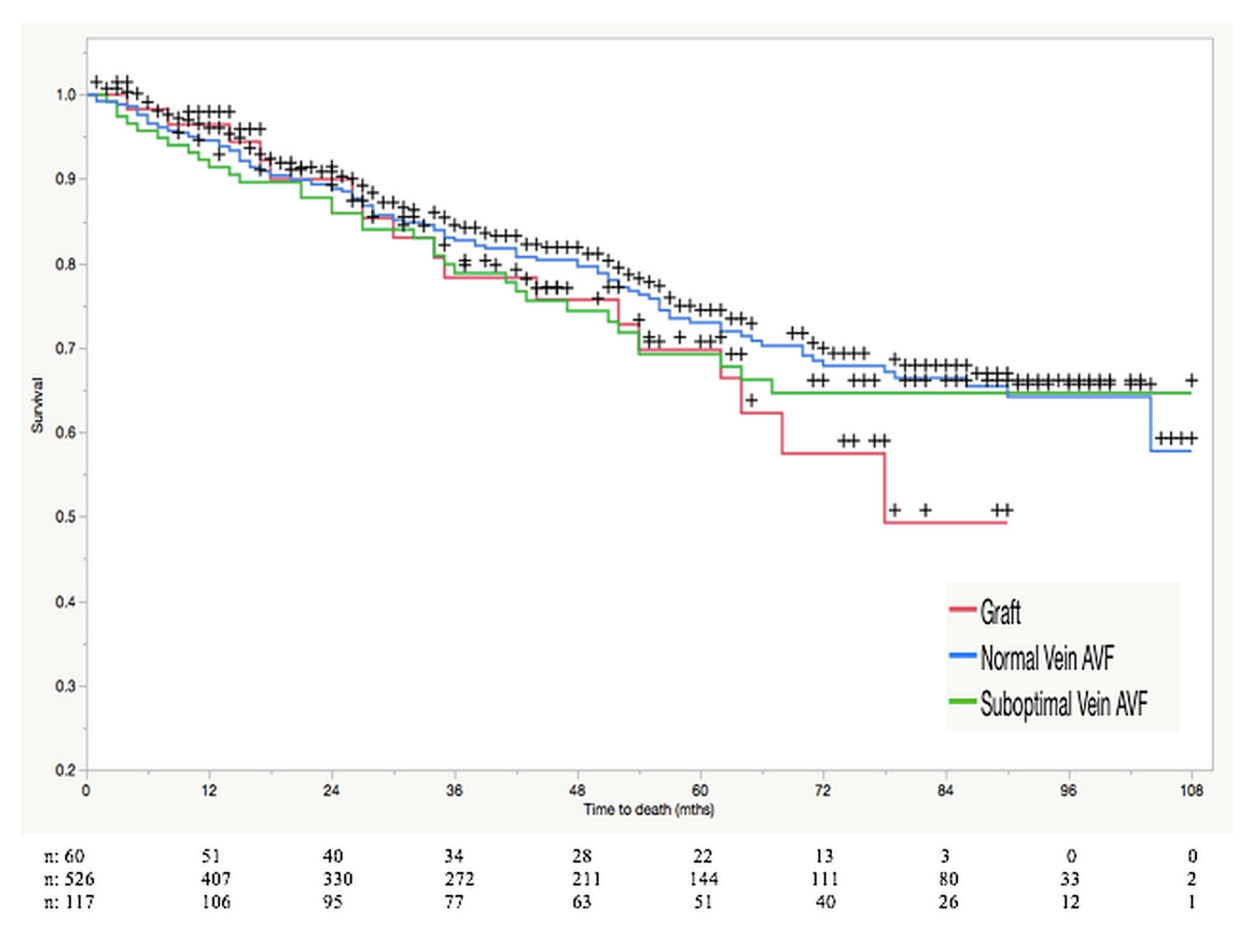
Kaplan-Meier analysis of survival rates among patients with various types of hemodialysis access Number of patients at risk (n) in each group is presented in the graph. Survival rates are shown as percentages at various points in time (months). P values calculated with the log-rank test. + signifies censored data point

A Kaplan-Meier statistical analysis comparing the primary assisted patency rates (Figure 1), as well as patient survival (Figure 2), among our three patient groups, was performed. Standard vein AVFs had a one and four-year primary assisted patency rate of 89.7% and 83.4%, respectively. This was in comparison to the one and four-year rate of 72.3% and 56.5% seen in the suboptimal vein AVF group and the rate of 62.4% and 48% seen in the AVG group, respectively. The primary assisted patency rates of each of the three groups at six months, one year, two years, three years, and four years are outlined in Table 1. Hazard ratios for occluded access were calculated and are reported in Table 2. Utilizing a log-rank test, we found the primary assisted patency rates to be significantly higher at all five time-points amongst our patients receiving AVFs in standard veins when compared to our suboptimal vein AVF and AVG groups (P<0.0001). When comparing the suboptimal vein AVFs to the AVG group, there was no significant difference in patency (P>0.05). Patient survival (Table 3) was comparable in all three groups. Survival rates for all three groups ranged between 91.4% and 96.5% after one year and between 75.6% and 79.7% after four years. Using the log-rank test (Table 4), no statistically significant difference was found between the groups (P>0.05).

**Table 1:** Comparing primary assisted patency rates among patients with various types of hemodialysis access Patency rates are shown as a percentage at six months, one year, two years, three years, and four years. N signifies the number of times that particular type of hemodialysis access was performed. * signifies a statistically significant difference between groups 1 and groups 2 and 3. AV: arteriovenous; AVF: arteriovenous fistula

Group	N	6 months	1 year	2 year	3 year	4 year
Standard Vein AVF	551	91.6%*	89.7%*	85.8%*	85.3%*	83.4%*
Suboptimal Vein AVF	131	73.4%	72.3%	68.2%	59.7%	56.5%
AV Graft	69	71.4%	62.4%	59.9%	57.6%	48.0%

**Table 2:** Hazard ratio for occluded access based on hemodialysis access type AV: arteriovenous; AVF: arteriovenous fistula

Treatment	Control	Risk Ratio	Prob>Chisq
Suboptimal Vein AVF	AV Graft	0.8239666	0.4568
Standard Vein AVF	AV Graft	0.2776355	< 0.0001
Standard Vein AVF	Suboptimal Vein AVF	0.3369499	< 0.0001
AV Graft	Suboptimal Vein AVF	1.2136415	0.4568
AV Graft	Standard Vein AVF	3.6018449	< 0.0001
Suboptimal Vein AVF	Standard Vein AVF	2.9677998	< 0.0001

**Table 3:** Comparing survival rates among patients with various types of hemodialysis access Survival rates are shown as a percentage at six months, one year, two years, three years, and four years. N signifies the number of patients who underwent that particular type of hemodialysis access. AV: arteriovenous; AVF: arteriovenous fistula

Group	N	6 months	1 year	2 year	3 year	4 year
Standard Vein AVF	526	96.6%	94.6%	88.8%	82.8%	79.7%
Suboptimal Vein AVF	117	95.3%	91.4%	85.9%	78.9%	75.6%
AV Graft	60	97.4%	96.5%	90.0%	78.3%	75.7%

**Table 4:** Hazard ratio for death based on hemodialysis access type AV: arteriovenous; AVF: arteriovenous fistula

Treatment	Control	Risk Ratio	Prob>Chisq
Suboptimal Vein AVF	AV Graft	0.8543109	0.5998
Standard Vein AVF	AV Graft	0.7612845	0.3125
Standard Vein AVF	Suboptimal Vein AVF	0.8911094	0.5622
AV Graft	Suboptimal Vein AVF	1.1705341	0.5998
AV Graft	Standard Vein AVF	1.3135694	0.3125
Suboptimal Vein AVF	Standard Vein AVF	1.1221966	0.5622

## Discussion

Overall the fistulas in this study demonstrated patency rates that were higher than what has been historically reported [2]. Our findings suggest that we were able to successfully create and mature functional AVFs in a group of patients with a large proportion of diabetes mellitus and suboptimal vasculature. These approaches included: 1) primary balloon angioplasty (PBA) at the time of fistula creation with subsequent balloon angioplasty maturation (BAM) procedures; 2) the 4 Fr radial artery approach for initial balloon angioplasty maturation with suboptimal veins; and 3) routinely scheduled clinical surveillance [14-16]. Despite not performing as well as standard vein AVFs in regards to primary assisted patency, the patients with suboptimal vein AVFs experienced similar patency and survival rates as compared to patients receiving AVGs. These findings were not unexpected, as it has been noted throughout the literature that AVFs created using suboptimal vasculature are more likely to suffer from decreased patency rates [9,14-16]. However, the benefits of using AVGs instead of AVFs for diabetics and patients with suboptimal vasculature did not prove true in the current study, as the two groups had comparable findings. In fact, the patients with suboptimal vein AVFs displayed slightly higher patency and survival rates relative to the AVG group, although this was not statistically significant. Nevertheless, through the implementation of our methods, we were able to successfully create and mature 131 AVFs using suboptimal venous anatomy (<2 mm diameter, sclerotic, accessory, or occluded veins) with results that were comparable, if not better than AVGs. According to the 2019 KDOQI guidelines, only one observational study evaluating the role of intraoperative PBA as a technique to upgrade small-diameter veins during AVF creation in combination with sequential BAM has been conducted [2]. This study, along with other smaller studies that the authors found, reported similar positive results when using PBA at the time of fistula creation in order to upgrade small/suboptimal veins for AVF creation [14-16]. However, none of these studies has extended the use of PBA at the time of fistula creation to their entire study population, nor has any study followed as many patients, for as long as we did.

Diabetes is a common comorbidity associated with ESRD and, consequently, many patients on dialysis have diabetes as well [10]. Due to the deleterious effects that diabetes has on vascular anatomy, fistula creation becomes more difficult and is not always possible. The pathological consequences of diabetes such as calcifications and atherosclerotic changes seen in the radial and brachial arteries are often major deterrents for fistula creation. Additionally, changes seen in the veins due to a high rate of hospitalizations, blood sampling, and intravenous (IV) placements make fistula creation very difficult [10]. One study found diabetes to be a strong independent risk factor for the primary occlusion of AVFs. Furthermore, when diabetes was combined with a cephalic vein diameter of ≤2 mm, the risk of failed fistula maturation and primary occlusion was markedly increased. The nondiabetic patients with venous diameters of >2 mm showed a higher maturation rate (odds ratio, 9.572; P=.006) as well as a higher primary patency rate (hazard ratio, 0.283; P=.004) than those with diabetes and venous diameters ≤2 mm [17]. In the current study, we were able to create fistulas in 90.8% (682/751) of our patient population, which included a demographic of 43.8% diabetics (308/703), utilizing our treatment methods. These findings contradict the notion that fistulas are difficult to create in diabetic patients and suggest that clinicians should keep an open mind when deciding HD access type in this population. This study was not the first to report high fistula creation rates in a large diabetic population. Golebiowski et al. demonstrated high AVF creation rates (94%) in a group of 166 diabetic patients, however, we were the first to do so on this large of a scale [10].

When selecting fistula location in this group, the pathological changes seen in the distal vasculature of diabetics have led many vascular surgeons toward electing to perform upper arm over forearm fistulas for initial HD access. Although both forearm and upper-arm AVFs offer a lot of the same benefits, forearm AVFs offer additional advantages, such as preserving upper arm veins for future use, less cosmetic displeasure, and decreased incidence of infection, heart insufficiency, and steal from the hand [10-11]. Several studies have demonstrated that arteries at or above the elbow region should be used for primary access because they usually provide the best quality artery in the diabetic population and thus allow for the highest chance of successful native AVF creation [11-13]. One study reported lower patency rates and an increased chance of immediate failure amongst diabetic patients undergoing forearm AVFs due to a high calcified burden in the forearm arteries [11]. They argue that this pattern of wall calcification is much less likely to occur in the brachial artery, leading them to suggest that brachiocephalic fistulas be considered for primary access, despite their disadvantages. Additionally, studies have noted that amongst diabetics, forearm AVFs are more likely to require additional procedures and longer periods of temporary catheter use, thus raising the risk of potential complications [12]. Utilizing the approaches outlined in our study, we were able to perform high rates (80.4%) of forearm (radiocephalic) fistulas in a population comprised highly of diabetics (43.8%). This is significant because it allows patients, who may previously not have been considered amendable to forearm AVF creation using standard approaches, to now receive all their stated benefits. These findings suggest that we may need to rethink deferring to upper-arm AVFs in diabetic as the standard approach moving forward.

Some of the disadvantages of fistulas that repeatedly come up in the literature are that fistulas have a higher rate of early failure, take longer to use, and do not always mature into usable vascular access and thus require subsequent revisions and additional procedures [15-16,18]. Intraoperative or immediate postoperative PBA during native AVF creation is a relatively newer method to deal with these issues and has proven to be a good salvage procedure for newly created, flow-limiting AVFs that are at high risk for failure. Despite concerns that intraoperative interventions may disturb the anastomosis site, studies have yielded strong patency rates when using these methods in high-risk patients with weak thrills [14-16,18]. BAM procedures are another means by which surgeons can improve and expedite AVF maturation [19]. One study demonstrated that maturation rates of fistulas created using small veins can be increased from 24.6% to 75.9% utilizing BAM [20]. However, BAM procedures are currently a controversial topic due to mixed results in the literature and the thought that it may actually promote AVF failure by causing endothelial and smooth muscle injury, which could promote neointimal hyperplasia [21]. One retrospective study comparing maturation times for AVF with and without using BAM in 194 patients found that there was no significant difference (average of 119 days vs 146 days, P=0.73). However, unlike the current study, they only focused on BAM in the postoperative period and did not address PBA at the time of AVF creation to upgrade small caliber veins [22]. Despite the discrepancies in BAM’s utility, our study yielded excellent patency and low AVF failure rates in the 643 patients who all underwent at least one BAM. Additionally, the use of micro-puncture access into the radial artery with a 4 Fr sheath and low profile system for the initial BAM in patients with suboptimal veins is a novel approach. To our knowledge, we are the first study to report the use of this method during our AVF maturation process. Our study demonstrated positive results in the 117 patients who underwent this particular procedure.

During our study, additional steps in the treatment of our patients were taken, which contributed to our excellent results. One of these was a clinical evaluation as well as an intervention, if needed, prior to AVF or AVG failure in all our patients. This included interventions such as thrombectomy, recanalization, aneurysm resection, coil embolization, and excision of infected grafts. Our clinical evaluations involved a thorough physical examination of the fistulas inflow, usable segment, and outflow observing for pulsatility, weakness, reasons for extravasation, and thrill. We also used intravascular ultrasound when indicated, for any patients with a contrast allergy, to evaluate for fistula stenoses. Along with our clinical assessments, we utilized techniques in our patients such as PBA at the time of fistula creation, BAM, accessory vein AVFs, mid-forearm AVFs, and recanalization of sclerotic veins. Lastly, a strong effort was made to reduce the time from fistula creation to maturation in an attempt to shorten the duration of indwelling catheters and lower complication rates.

## Conclusions

Utilizing our treatment approaches, we were able to achieve a high AVF creation rate amongst a population with a large proportion of patients with suboptimal veins and comorbidities such as diabetes mellitus. We demonstrated high four-year patient survival and patency rates, all while attempting to reduce maturation times and decrease the duration of indwelling catheters. In the 117 patients with suboptimal veins, functional lower-arm fistulas were created and successfully matured using an initial 4 Fr radial artery approach with four-year assisted patency rates slightly worse than standard vein AVFs but patency and patient survival rates that were comparable to patients with AVGs. Through the use of all of these approaches outlined in this study, we have demonstrated an ability to salvage suboptimal veins for fistula creation, which enhances our potential for creating AVFs in a group of patients who would otherwise not be amenable to fistula creation.
